# Blood transfusion requirements among children with severe malarial anemia: a cross-sectional study in a second level reference hospital in Burkina Faso

**DOI:** 10.11604/pamj.2020.37.108.22384

**Published:** 2020-10-01

**Authors:** Salam Sawadogo, Koumpingnin Nébié, Tieba Millogo, Eléonore Kafando

**Affiliations:** 1 Joseph KI-ZERBO University, Ouagadougou, Burkina Faso,; 2 African Institute of Public Health, Ouagadougou, Burkina Faso

**Keywords:** Malaria, anemia, blood transfusion, rationale use of blood, blood requirement

## Abstract

**Introduction:**

blood transfusion (BT) is an important part of pediatrics healthcare in sub-Saharan Africa because of anemia due to malaria, malnutrition and hereditary anomalies of red blood cells. However, BT services experienced chronic blood shortage, unsafe blood products and poor procedures of clinical use of blood. This results in inadequate management of severe anemia.

**Methods:**

to assess the quality of BT requirements in severe malarial anemia at the regional hospital center of Koudougou in Burkina Faso, we carried out a cross-sectional study including 402 children with severe malaria (WHO 2000 criteria).

**Results:**

over the study period, severe malaria represented 45.6% (402/882) of pediatric admissions. Anemia was observed in 97.5% (392/402) of cases and BT was required for 78.4% (315/402). The median age was 16 months (IQR 9-27) and the average hemoglobin was 51.4±22.2 g/L. The prescriptions were in accordance with WHO and national guidelines respectively in 63.8% and 92.7%. Blood units were issued in 99.4% (350/352) of blood orderings. Out of 350 blood units delivered, blood was administered in 98% (343/350). The median actual time to transfusion was 65 minutes (IQR: 45-100) and median transfusion duration was 73.8 minutes (IQR: 47.5-110). The signs of intolerance to anemia disappeared in 134/138 cases (97.1%) and the average haemoglobin increased of 37.9±17.6 g/L. Death occurred in 23 cases (5.7%).

**Conclusion:**

the management of severe malaria requires blood transfusion in almost half of cases. Blood was available to meet most requests. However, efforts are still required for proper use of the blood.

## Introduction

Blood transfusion (BT) is a therapeutic commonly used in sub-Saharan Africa, since severe anemia remains a public health problem. Indeed, severe anemia accounts for 9.7 to 29% of pediatric admissions and 8 to 17% of hospital deaths [1-3]. The common cause of severe anemia was severe malaria associated or not to malnutrition, hereditary abnormalities of red blood cells (RBCs) [4,5]. Its appropriate management involves BT in order to correct promptly hemoglobin (Hb) level. But, BT in these countries remains a big concern. Indeed, the difficulties to make available timely safe transfusion do not allow adequate management of severe anemia, leading to lethality up to 5-10%, with 25-50% occurring within 6 hours of admission [6,7]. In order to address the major gap between blood supply and demand, the strategy of the World Health Organization (WHO) recommends the reduction of unnecessary transfusions. The WHO guidelines indicate BT in children with a Hb <40g/L, or in case of Hb between 40 and 60g/L associated with life-threatening complications such as respiration distress or impaired consciousness [8]. But the compliance to these recommendations is often poor and many children receive unwarranted transfusions [9]. In Burkina Faso, malaria is the main reason for hospitalization and first cause of in-hospital mortality among children [10]. The national guidelines for malaria management recommends BT in cases of Hb <50g/L with clinical intolerance signs to anemia [11]. A study evaluating the conformity of malaria management concluded that the guidelines were not complied [12]. There are scarce data on BT in children in our context. Also, our study aims at assessing the quality of transfusion requirements among children with severe malarial anemia, in a regional hospital in Burkina Faso.

## Methods

**Study setting:** we conducted the study in the regional hospital center of Koudougou (CHR-K), in a semi-urban city of the central-west region with 374,373 inhabitants in 2016 [13]. The climate is characterized by a rainy season from June to September and dry one from October to May. Malaria transmission is markedly seasonal and intense during the rainy season [14]. CHR-K was a 190-beds second-level hospital with an hospitalization turnover around 15,000 per year [13]. It serves as a reference hospital for seven district hospitals. In 2016, malaria accounted for 49.5% of medical consultations and severe malaria 20.1% of the total hospitalizations and 14.8% of deaths in central-west region [13].

**Study design:** a total of 402 consecutive children aged 0 to 14 years, admitted in pediatric ward between September and November 2016, with severe malaria according to the WHO criteria defined in 2000 [15] were included in a cross-sectional study. Clinical and biological parameters were collected within 24 hours from admission. Trained investigators recorded socio-demographic information, history of symptoms and pre-admission treatments. Physical examination, including vital signs, were performed as part of the hospital standard cares. Data about blood prescription and administration were collected from clinical records and post-transfusion and hemovigilance forms.

**Laboratory procedures:** the diagnosis of malaria was made by trained microscopist using blood smear test. Pre- and post-transfusion Hb was measured as part of the complete blood count using automated hematological analyzer (BC3000-plus® PLC, MINDRAY Corporation Chenzen, China). Additional biochemical analyzes (glucose, urea, creatinine, serum electrolytes) performed for some children by treating physician were recorded. A double determination of ABO/RHD blood group was performed prior to transfusions. Screening for irregular antibodies, 37°C cross-match, direct antiglobulin test and reticulocyte count were not performed.

**Malaria management and blood transfusion procedures:** malaria cases were managed according to the 2014 national guidelines [11]. BTs were provided at the discretion of treating physician. The need for transfusion was based on laboratory-confirmed Hb <50g/L or on the presence of clinical intolerance signs [11]. Blood components were supplied by the regional blood transfusion center of Koudougou (CRTS-K) located at 5 minutes from pediatric ward. Blood was collected from unpaid voluntary donors. HIV, viral hepatitis B and C and syphilis were systematically screened on all blood units using 4^th^ generation ELISA tests. The main blood component processed was the red cell concentrates (RCC). It was produced by settling whole blood by simple gravity as previously described [16]. Fresh whole blood (collected and screened within 6 hours) was also processed on clinician request. Blood was issued free of charge to patients on medical request stated on a standard prescription form. ABO/RHD blood group and room temperature cross-match against recipient serum were checked before blood delivery. Blood was transported from the distribution service to the patient's bed by healthcare unspecific support agents. The prescribed volume was weight-based (10-15mL/kg). When the delivered volume was higher than prescribed volume, the actual volume administered was estimated based on blood unit size. Children were monitored throughout and after the transfusion. Immediate clinical outcomes and Hb level were assessed respectively within 4 hours and 24-36 hours post-blood administration.

### Study definitions

**Anemia and non-tolerated anemia:** according to the WHO guidelines, age-dependent non-tolerated anemia cases were defined as Hb <115g/L among children under 59 months or <110g/L among 5-11 years children or <120g/L among 12-14 years ones with one or more of following signs: asthenia, tachycardia, tachypnea, shock, dehydration, impaired consciousness, respiratory distress and heart failure [17].

**Severe malnutrition:** severe acute malnutrition was stated as weight-for-height z-score under -2 SD for age and sex or presence of bilateral lower limb edema [17].

**Quality of blood transfusion practices:** we assessed the quality of blood transfusion practices against the following criteria:

**The compliance with the WHO and the 2014 national guidelines:** according to WHO guidelines, transfusion is indicated for children with Hb <40g/L, or between 40 and 60g/L associated with life-threatening complications (respiratory distress, cardiac failure, dehydration, shock, impaired consciousness) [8]. The national guidelines recommend transfusion if Hb <50g/L or in the presence of clinical intolerance signs [11].

**The management of critical emergencies:** the national guidelines for good practices in BT [18] defined three emergency levels: immediate vital emergency where blood must be delivered without delay; vital emergency where blood must be delivered within 30 minutes and relative emergency where blood can be delivered beyond 2 to 3 hours. In the current study, we defined two emergency levels: immediate life-threatening emergency (LTE) requiring BT within 30 minutes; relative emergency (ER) requiring BT beyond 30 minutes. The ability of healthcare and BT services to treat a LTE blood request was assessed by: the “actual time to transfusion” defined as the interval time from blood prescription to the transfusion start time. It includes 3 components: the “blood ordering delivery time” (from blood prescription to blood ordering reception at the blood service) which is the responsibility of the supportive healthcare agents; the “blood issue time” (from ordering reception to the blood delivery) which depends from nurses and laboratory technicians of the blood service; the “in-service blood conservation time” (from blood issuing to transfusion starts) which depends from nurses in healthcare wards. The “transfusion duration” was the time between the start and end of BT which also depends from nurses in healthcare wards. The efficacy of blood transfusion assessed clinically (disappearance of signs of intolerance) and biologically (increase in hemoglobin).

**Data analysis:** data were analyzed using STATA-13. Mean ± 2 SD (standard deviation) and median with interquartile range (IQR) were used to describe numerical variables and proportion for categorical variables. In univariate analysis comparing children who received blood prescription to whom who did not, we used Mann-Whitney test, student t-test, Chi-square or Fisher exact tests as appropriated. The significance level of the different tests was p <0.05.

**Ethical considerations:** the study was approved by the medical and scientific committees of both CHR-K and national blood transfusion center. Informed consent of children´s mother or father was obtained prior to inclusion in the study. They were informed that the quality of care was not dependent on their consent or not to participate in the study. Anonymity and confidentially were guaranteed for all participants. Results of biological tests performed were used by treating physician for patient management.

## Results

**Baseline characteristics:** over our study period, 402 children (45.6%) of 882 hospitalized were included. From which, 392 (97.5%) presented with anemia and BT was required for 315 (78.4%). The median age of these 315 children was 13 months (IQR: 9-24) and 37.8% were under 12 months. The average Hb was 51.4±22.2g/L and microcytosis was found in 41.8%. The Table 1 and Table 2 summarize the socio-demographic, clinical and biological characteristics of the 402 children included.

**Table 1:** socio-demographic characteristics of the 402 children with severe malaria and the 315 for whose blood products have been ordered, Regional Hospital of Koudougou, Burkina Faso, 2016

Parameter	Total (N=402)	Blood ordering		Odd-ratio	[95%CI]	p-value
		Yes (N=315)	No (N=87)			
	n; (% column)	n; (% row)	n; (% row)			
**SexΦ**						
Male	214 (53.2)	169 (79.0)	45 (21.0)	1	-	-
Female	188 (46.8)	146 (77.7)	42 (22.3)	0.9	0.6-1.5	0.75
Age in months (median; IQR)þ	16 [9-27]	13 [9-24]	30 [18-48]	44.5	-	0.0001
**Age group (months)Φ**						
<12	132 (32.8)	119 (90.2)	13 (9.8)	1	-	-
12 – 23	121 (30.1)	106 (87.6)	15 (12.4)	0.8	0.3-1.7	0.5
24 – 35	64 (15.9)	44 (68.7)	20 (31.3)	0.2	0.1-0.5	<0.001
36 – 47	36 (9.0)	20 (55.6)	16 (44.4)	0.1	0.1-0.3	<0.001
48 – 59	21 (5.2)	12 (57.1)	9 (42.9)	0.1	0.1-0.4	<0.001
≥60	28 (7.0)	14 (50.0)	14 (50.0)	0.1	0.0-0.3	<0.001
Residence in rural areaΦ	268 (66.7)	237 (88.4)	31 (11.6)	5.5	3.3-9.2	<0.001

Φ Chi-squared or Fischer test; þ Wilcoxon-Mann-Whitney; IQR = interquartile ranges

**Table 2:** clinical and biological characteristics of the 402 children with severe malaria and the 315 for whose blood products have been ordered, Regional Hospital of Koudougou, Burkina Faso, 2016

Parameter	Total (N=402)	Blood ordering				p-value
		Yes (N=315)	No (N=87)	Odd-ratio	[95%CI]	
	n; (% column)	n; (% row)	n; (% row)			
Onset of symptoms in day (mean ± sd) ǂ	3.2±1.6	3.3±1.7	3.0±1.5	-	-	0.06
Severe malnutritionᶲ	85 (21.1)	78 (91.8)	7 (8.2)	3.1	1.8-5.5	<0.001
Hemoglobin level in g/L (mean ± sd) ǂ	51.4±22.2	42.3±12.7	84.0±18.4	-	-	<0.001
**Hemoglobin level range (g/L)ᶲ**						
<40	132 (32.8)	132 (100.0)	0 (0.0)	1		-
40 – 60	160 (39.8)	153 (95.6)	7 (4.4)	58.3	24.5-138.6	<0.001
>60	110 (27.4)	30 (27.3)	80 (72.7)	1	-	-
Parasitic density ((mean ± sd) x 1000) ǂ	36.83±71.86	35.55±69.19	41.63±81.04	-	-	0.5
**Indicators of severe malariaᶲ**						
Cerebral malaria	16 (4.0)	10 (62.5)	6 (37.5)	0.4	0.1-1.2	0.1
Repeated convulsions	66 (16.4)	25 (37.9)	41 (62.1)	0.1	0.1-0.2	<0.001
Impaired consciousness	68 (16.9)	44 (64.7)	24 (35.3)	0.5	0.3-1.0	0.003
Prostration	72 (17.9)	50 (69.4)	22 (30.6)	0.4	0.2-0.7	0.04
Respiratory distress	51 (12.7)	44 (86.3)	7 (13.7)	1.8	0.8-4.3	0.1
Shock	12 (3.0)	11 (91.7)	1 (8.3)	3.1	0.4-24.4	0.3
Abnormal bleeding	1 (0.2)	1 (100.0)	0 (0.0)	1	-	-
Macroscopic hemoglobinuria	21 (5.2)	18 (85.7)	3 (14.3)	1.7	0.5-5.9	0.4
Clinical jaundice	26 (6.5)	14 (53.8)	12 (46.2)	0.3	0.1-0.6	0.003
Severe anemia	261 (64.9)	261 (100.0)	0 (0.0)	1	-	-
Renal impairment	5 (1.2)	3 (60.0)	2 (40.0)	0.4	0.1-2.5	0.3
Hyperparaitemia	21 (5.2)	16 (76.2)	5 (23.8)	0.9	0.3-2.5	0.8
Hypoglycemia	27 (6.7)	17 (63.0)	10 (37.0)	0.4	0.2-1.0	0.04
In-hospital length of stay (day) (mean ± sd) ǂ	3.3±2.2	3.5±2.3	3.3±2.0	-	-	0.9
Deathᶲ	23 (5.7)	17 (73.9)	6 (26.1)	0.8	0.3-2.0	0.6

ǂ ANOVA; ᶲ Chi-squared or Fischer test; g=gramm; L=liter; sd=standard deviation

**Blood products ordering:** a total of 352 blood orderings were issued for the 315 children. In 43.8% of children, the indication was the presence of intolerance signs to anemia, with tachypnea in 58.1% of cases, tachycardia (54.7%), asthenia (29.1%), impaired consciencious (10.1%). Blood was ordered because of an Hb <40g/L only in 21.9% and between 40 and 60g/L in 29.8%. In 4.4% of cases, blood was ordered despite an Hb >60g/L. WHO and national guidelines were complied respectively in 63.8% and 92.7%. Out of the 352 blood ordering, the emergency degree was stated on 342 forms. In 54.4% (186/342), it was stated as LTE.

**Blood issue and administration:** in 99.4% (350/352), blood units were issued. ABO identical-phenotype blood was delivered in 88.9% versus non-identical-phenotype units in 10.5%. In two (0.6%) blood requests for O negative units, no blood unit was delivered. In 2% of cases, the volume delivered was less than 50% of requested volume, more than 80% of requested volume in 78.7% of cases. Out of the 350 deliveries, blood was administered in 98% (343/350). The median actual time to transfusion was 65 minutes (IQR: 45-100) and was associated (Table 3) to the presence of non-tolerated anemia (p=0.005). When considering the different sequences of this actual time to transfusion (Table 3), only in-service blood conservation time was significantly associated to the degree of emergency and the presence of non-tolerated anemia. With a median of 73.8 minutes (IQR: 47.5-110), transfusion duration was associated to the degree of emergency (Table 3).

**Table 3:** comparison of different median times for transfusion management in children with malaria anemia (N=315) according to certain emergency criteria at the regional hospital in Koudougou, Burkina Faso, 2016

	Actual time to transfusion (min)		Blood ordering delivery time (min)		Blood issue time (min)		In-service blood con-servation time (min)		Transfusion duration (min)	
	Median [IQR]	p-value	Median [IQR]	p-value	Median [IQR]	p-value	Median [IQR]	p-value	Median [IQR]	p-value
Overall	65 [45-100]	Na	20 [11-42]	na	14 [10-20]	na	25 [15-45]	na	73.8 [47.5-110]	na
**Degree of emergency**										
LTE	63 [45-90]	0.471	20 [11-37]	0.196	15 [10-20]	0.604	22 [14-38]	0.004	72.8 [49-105]	0.043
RE	75 [53-105]		20.5 [13-48]		13 [10-20]		30 [18-50]		76 [46-115]	
**Importance of anemiaϷ**										
< 40 g/L	64 [44-100]	0.310	19.2 [11-40]	0.127	12.5 [10-20]	0.576	24 [15- 44]	0.478	80 [50.5-111]	0.144
40 - 60 g/L	72 [53-100]		25 [14-45]		15 [10-20]		25 [15-45]		72 [46-110]	
>60 g/L	61.5 [45-90]		20 [5-38]		13 [9-20]		20 [15- 38.5]		67.5 [42.5-85]	
**Non-tolerated anemia**										
Yes	60 [45-81.5]	0.005	20 [13-35.5]	0.279	14 [10-20]	0.857	20 [14-31]	0.000	70 [45-100]	0.051
No	75 [50-110]		20 [1150]		13 [10-20]		30 [18- 50]		80 [51-110]	

LTE = life-threatening emergency; RE = relative emergency; IQR = inter-quartile range; min = minute; g = gramm; L = liter; na = non applicable; Ϸ: Kruskal-Wallis test. In other cases, Wilcoxon-Mann-Whitney test was used

**Clinical and biological outcomes:** the signs of intolerance to anemia disappeared in 134/138 cases (97.1%). The average post-transfusion Hb measured in 283 patients was 80.1±16.2 g/L (range: 45-140). The average increase of Hb was 37.9±17.6 g/L (range: -4 to 104). The average of in-hospital length of stay was 3.3±2.2 days (Table 1). Deaths occurred in 5.7% and in 3.7% patient were discharged against medical advice. For two deceased children, compatible blood unit was not found in one case and for the other, death occurred before transfusion began.

## Discussion

To the best of our knowledge, this is the first report on this topic. Unlike other studies in sub-Saharan Africa [2,19-21], our study was conducted in a context of substantial improvement of blood products availability and clinical practices. The transfusions from relatives blood donations were no longer practiced in many healthcare facilities [22]. But our study has some limitations. It was a single hospital-based study performed over a limited period; thus the study may lack strength. In addition, poor laboratory analysis capacities may had limited the ability of differential diagnostics and delayed or induced mistreatment. This could explain low post-transfusion Hb recovery in some cases. Thirdly, since we did not use a time stamp system, the different times was written manually. So, major approximations could occur and affect the accuracy of calculations. Despite these limitations, the study demonstrates the need of improvement of the quality of BT in our country. We highlighted important areas for intervention, namely at the clinical-blood bank interface. The in-hospital prevalence of severe malaria (45.6%) was more than the double of the prevalence reported in the centre-west region (20.1%) and 11% higher than that reported at national level (34.7%) [13].

This difference is due to the fact that our study was conducted during a period of high seasonal malaria transmission [14]. About 97.5% of children with malaria were anemic, 64.9% had severe anemia (Hb <50 g/L) and 34.3% had life-threatening anemia. We found a higher rates of malarial anemia and life-threatening anemia than in Kenya (respectively 72% and 29.5%) [21]. It is probably not fair to attribute all these anemia cases to malaria. Certainly, plasmodium causes hemolysis, all the more important as the parasitaemia is high, but anemia´s in sub-Saharan Africa have multifactorial causes, including nutritional deficiencies, hereditary RBCs abnormalities, parasitic and bacterial infections. Incomplete laboratory investigations have limited our ability to distinguish anemia due to hemolysis and those resulting from dyserythropoiesis.

Nonetheless, we can speculate that the microcytosis (41.8%) observed in our study could be attributed to iron deficiency and/or alpha-thalassemia that are common in sub-Saharan area [4,23,24]. The frequency of anemia and high requirement for BT highlight the need for minimal laboratory diagnostic capacity, adequate supply of pathogen-free, quality-assured blood products and appropriated clinical use of blood in resource-limited healthcare facilities. Unfortunately, many studies showed that equipment for Hb measurement, blood supply chain and skilled healthcare staffs may be lacking in low-income settings, resulting in transfusion delays [20,21,25] and inappropriate use of blood [26,27].

In our study, blood orderings did not fulfilled the WHO and 2014 national criteria for BTs respectively in 32.2% and 7.3%. This is lower than the 46% of inappropriate blood transfusions in Kenya [27]. National guidelines that recommends BT in case of Hb <50g/L or in cases of non-tolerated anemia [11] seem to be “liberal” compared to WHO guidelines. Such a “liberal” transfusion threshold should allow nurses (the patient´s first contact in hospital) to order blood and thus, compensating for the understaffing in physicians that does not allow close-clinical monitoring of patients to decide timely for BT. The assumption was that patients in worse clinical condition should not survive to wait for physician´s clinical assessment and the blood donation from compatible relatives. Some African studies reported that delays in getting blood was one of the worst issues affecting patients survival [25]. In Nigeria, authors noted that the children who did not receive BT while required it, died within 2 hours at admission [28]. In this current study, the median (IQR) actual time to transfusion was 65 (45-100) minutes. It was similar to that noted in Malawi (1 hour) [29] but shorter than that reported in Republic Democratic of Congo (4 hours) [2], in Kenya (3.3 to 6.7 hours) [21,27] and in Tanzania (7.8 to 21 hours) [3,19]. The median actual time for BT beginning was significantly shorter in case of life-threatening anemia (60 versus 75 min; p=0.005). But this time remained much longer than that is recommended in the national guidelines (<30 minutes). The sequences which prolong the time to make blood available for transfusion were the blood ordering delivery time (median of 20 minutes) and the in-service blood conservation time (median of 25 minutes). The in-service blood conservation time was shorter when the physician stated his request as LTE (p=0.004) or when the patient did not tolerated his anemia (p <0.0001). The supportive healthcare agents ensuring that the prescription should be sent to the transfusion service have not always necessary skills to distinguish urgent requests. In addition, they were assigned to other tasks and were not always at sufficient numbers. All this can delay the delivery of blood ordering to the transfusion service. On the other hand, the nurses in the care services were assumed to be skilled for proper and safe BTs. The blood service did not made any discrimination between blood requests. The median blood issue time (14 minutes) was the same, whether the transfusion was requested as LTE or not. This is very worrying, if considering that blood services are supposed to have the best trained agents. A more general reason for these shortcomings could be the absence of systematic procedures for managing LTE transfusions, both in the hospital and transfusion service. Despite these insufficiencies, our findings suggest a more well-structured blood supply chain in our settings as previously mentioned [30]. Indeed, unlike to certain countries, in Burkina Faso, a centralized BT system was put in place since 2000. The short blood issue time indicates that, contrary to some settings where blood stock-out was frequently reported [2,20,21,25], in our context, blood were more available. The quantitative satisfaction rate of blood demand was 99%. We found that 90.5% of children improved and death occurred in 5.7%. The death rate was lower than in Nigeria (13.6%) [28], but similar to that reported in RD Congo (5.6%) [2], in Kenya (5.6% and 7%) [21,31]. This difference could be explained by differences in study participants and the availability of blood.

## Conclusion

Our study confirms the high requirement of BT among children with severe malaria. Blood was most often available to meet the majority of demands. However, the few unmet requests have resulted in fatal outcomes in children. In addition, high proportion of transfusions did not complied guidelines. Therefore, the efforts of transfusion services should aim at developing transfusion counseling to guide prescribers towards a rational use of blood and avoid unnecessary transfusions. Another aspect should be the strengthening of measures to reduce blood demands by addressing malaria, the most common cause of severe anemia. All these efforts should allow the optimization of blood availability and the quality of its use for children which really need transfusion.

### What is known about this topic

- Malarial anemia was the main reason for blood transfusion;
- High rate of unnecessary and unwarranted blood transfusion;
- Important delays in blood transfusion because of blood shortage.

### What this study adds

- National guidelines for blood transfusion were not complied;
- Delays in blood transfusion were not only imputable to blood shortage, but also to the insufficiency in services organization;
- Need for implementing transfusion counselling for clinicians in our context.
